# A consistent and corrected nighttime light dataset (CCNL 1992–2013) from DMSP-OLS data

**DOI:** 10.1038/s41597-022-01540-x

**Published:** 2022-07-20

**Authors:** Chenchen Zhao, Xin Cao, Xuehong Chen, Xihong Cui

**Affiliations:** 1 State Key Laboratory of Remote Sensing Science, Faculty of Geographical Science, Beijing Normal University, Beijing, 100875 China; 2 Beijing Engineering Research Centre for Global Land Remote Sensing Products, Faculty of Geographical Science, Beijing Normal University, Beijing, 100875 China

**Keywords:** Socioeconomic scenarios, Sustainability, Energy management

## Abstract

Remote sensing of nighttime light can observe the artificial lights at night on the planet’s surface. The Defense Meteorological Satellite Program’s Operational Line Scan (DMSP-OLS) data (1992–2013) provide planet-scale nighttime light data over a long-time span and have been widely used in areas such as urbanization monitoring, socio-economic parameters estimation, and disaster assessment. However, due to the lack of an on-board calibration system, sensor design defects, limited light detection range, and inadequate quantization levels, the applications of DMSP-OLS data are greatly limited by interannual inconsistency, saturation, and blooming problems. To address these issues, we used the power function model based on pseudo-invariant feature, the saturation correction method based on regression model and radiance-calibrated data (SARMRC), and the self-adjusting model (SEAM) to improve the quality of DMSP data, and generated a Consistent and Corrected Nighttime Light dataset (CCNL 1992–2013). CCNL dataset shows good performance in interannual consistency, spatial details of urban centers, and light blooming, which is helpful to fully explore the application potentials of long time series nighttime light data.

## Abstract (summary)

**Table:** 

Measurement(s)	nighttime light intensity
Technology Type(s)	satellite radiometer

## Background & Summary

Remote sensing of nighttime light (NTL) can detect weak artificial light at night and obtain surface information completely different from that in the daytime, so it is widely used to monitor various information and changes related to human activities^1,2^. In recent years, there have been some remote sensing satellites of nighttime light and the data products, such as Luojia-1, JL1-3B, etc^3,4^. However, the human activity progress reflected by the change of NTL in a long time series can only rely on the Defense Meteorological Satellite Program’s Operational Line Scan (DMSP-OLS) data available since 1992 and the Suomi National Polar-orbiting Partnership’s Visible Infrared Imaging Radiometer Suite (NPP-VIIRS) data available since 2012. The long history of temporal NTL data is widely used to study the urbanization process^5^, demographic changes^6^, economic changes^7^, power consumption^8^, and other research. Therefore, obtaining high-quality NTL data for research on the change in these long-term human activities is of great significance.

The stable lights dataset from Version 4 DMSP-OLS Nighttime Lights Time Series is the most common and most commonly applied DMSP-OLS data product. However, they suffer from the problems of interannual inconsistency, saturation, and blooming^9–13^. (1) Interannual inconsistency. DMSP-OLS NTL annual composite from 1992 to 2013 is acquired by sensors onboard six different satellites without calibration mechanism^14^. The lack of onboard calibration, sensor degradation, and satellite orbit drift result in interannual inconsistency for the sum of NTL digital number (DN) values at global and regional scales^15^. (2) Saturation. Since stable light products are acquired under low moonlight illumination conditions, the sensors need to be set at a high gain to detect weak ground light, which leads to oversaturation problems in areas of high brightness, especially in urban centres^16^. Due to the 6-bit quantization and low dynamic range of OLS data, the DN value no longer increases with the increase of ground light intensity when it reaches 63. (3) Blooming. The possible reasons for the blooming effect can be summarized as sensor field of view changes during scanning, accumulation of geographic bias in data synthesis, data resampling during onboard data storage, and atmospheric effect^12,17,18^.

Researchers have proposed several methods to address these problems. For the interannual inconsistency of the DMSP-OLS stable light data, one of the common relative calibration methods is the pseudo-invariant feature (PIF)^19^. Elvidge *et al*.^14^ took Sicily as a PIF region and F12-1999 as the reference image and adopted the second-order polynomial function model to correct the interannual inconsistency of other images. Li *et al*.^20^ proposed a stepwise calibration method, which used prior knowledge to judge the anomalies in NTL time series curves and processed images from multiple sensors in turn. In response to the saturation problem of DMSP-OLS data, some studies have used auxiliary data such as vegetation index^21^, surface temperature^22^, and DMSP-OLS radiance calibrated data^23,24^ to restore light information in saturated areas. In eliminating the blooming effects of DMSP-OLS data, Abrahams *et al*.^25^ and Zheng *et al*.^18^ took the blooming effect as an image blur problem and that the blooming brightness of an image pixel can be fitted with a Gaussian surface. Cao *et al*.^26^ developed the self-adjusting model (SEAM) based on the spatial response function to correct the blooming effect without using other ancillary data. Zhuo *et al*.^27^ proposed an improved SEAM model (iSEAM) considering spatial heterogeneity of effective blooming distance while introducing land cover data. Based on the above methods, some global or regional NTL data products were generated to overcome one or more of these problems^14,28–31^. However, a global data product for all the solvable issues has yet to emerge.

Therefore, this study aims to address the three problems of interannual inconsistency, saturation, and blooming of DMSP-OLS stable light data and to produce a consistent and corrected nighttime light dataset (CCNL 1992–2013) from DMSP-OLS data. The CCNL dataset produced in this study will lay the foundation for creating complete sequence (1992-present) NTL data and provide valuable data for the applications of historical DMSP-OLS data.

## Methods

### Data collection

The datasets utilized in this study include two categories, as shown in Table 1. The first one generates the global consistent and corrected nighttime light (CCNL) dataset, including DMSP-OLS stable light product and the radiance calibrated nighttime light product. The second category is the auxiliary datasets to assess the quality and accuracy of the CCNL dataset, which contains other types of NTL products, urban land products, and socio-economic statistics.

**Table 1:** Datasets for production and evaluation of CCNL dataset.

Usage	Name	Type	Time step	Format	Authors
DMSP-OLS Data	Stable Lights product	NTL data	1992–2013	Raster data	Elvidge *et al*.^7^; Baugh *et al*.^32^
	Radiance Calibrated product	NTL data	1996, 1999, 2000, 2003, 2004, 2006, and 2010	Raster data	Elvidge *et al*.^16^; Hsu *et al*.^29^
Auxiliary Data	NPP-VIIRS	NTL data	2013	Raster data	Elvidge *et al*.^1^
	EANTLI	NTL data	2013	Raster data	Zhuo *et al*.^21^
	GAIA	Urban area	1990, 1995, 2000, 2005, 2010, 2015, and 2018	Vector data	Gong *et al*.^34^
	UrbanLand	Urban area	1992, 1996, 2000, 2006, 2010, and 2016	Raster data	He *et al*.^35^
	socio-economic data	Statistical data	1992–2013	Textual data	World bank

(1) **Stable light product**

Nighttime stable lights product is one of the datasets belonging to Version 4 of Global DMSP-OLS Nighttime Lights Time Series (1992–2013), which has been applied to various areas. It is a cloud-free annual composited product that collects all the available archived DMSP-OLS smooth resolution data for calendar years from six satellites, F10, F12, F14, F15, F16, and F18. The stable light products are composited cleaned up average visible band digital number values containing the lights from cities, towns, and other sites with fires have been discarded^7,32^. The background noise was identified and replaced with values of zero. Data values range from 1 to 63. Areas with zero cloud-free observations are represented by the value 255. The products are 30 arc-second grids, spanning −180 to180 degrees longitude and −65 to 75 degrees latitude. The product is free and available at https://eogdata.mines.edu/dmsp/downloadV4composites.html.

(2) **Radiance calibrated nighttime light product**

**Fig. 1:**
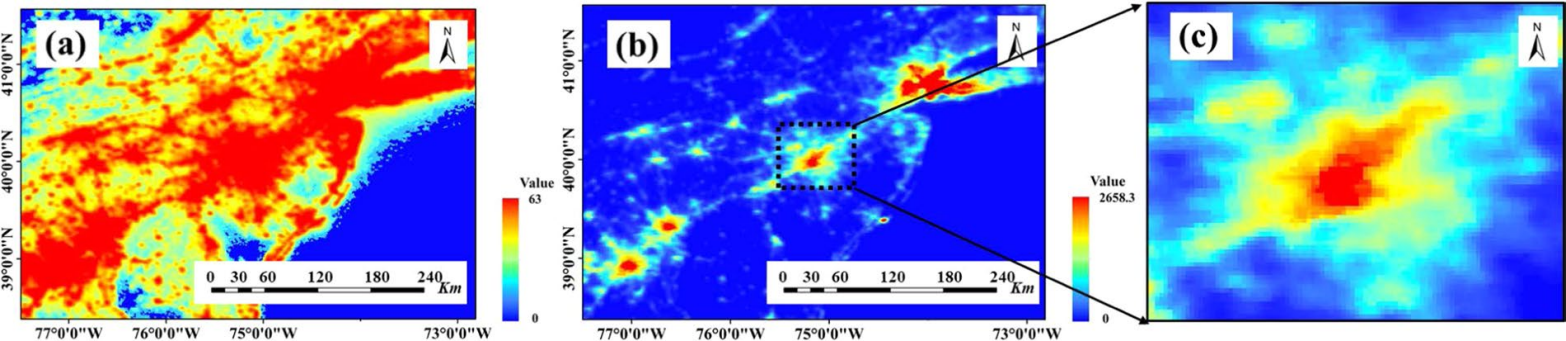
Comparison of stable light data and radiance calibrated data of US East Coast in 2010. (**a**) F182010, (**b**) F16_20100111–20101209_rad_v4, and (**c**) spatially amplified view of downtown Philadelphia from Radiation Calibrated data.

Global radiance calibrated nighttime lights were produced without sensor saturation by combining sparse data acquired at low gain settings with the operational data obtained at high gain settings which can be related to radiances based on the pre-flights sensor calibration^7,16,22^. This product has the exact resolution and coverage as the stable light product. Due to limitations in the acquisition of low gain data, the radiance calibrated product is only available in 7 different years (circus 1996, 1999, 2000, 2003, 2004, 2006, and 2010). The product is free and available at https://eogdata.mines.edu/dmsp/download_radcal.html. Radiation Calibrated data significantly eliminates the saturation effect by synthesizing data at different gains and has substantial advantages in spatial analysis. However, the number of images is relatively small to meet the requirements of some studies. Figure 1 shows that the radiance calibrated data portrays the change of brightness in the urban core than the stable light data, which is conducive to studying spatial and temporal changes within the city.

(3) **NPP-VIIRS**

Version 1 VIIRS Day/Night Band Nighttime Lights is a superior NTL dataset than DMSP-OLS data, mainly providing daily composites and monthly composites products since April 2014 (https://eogdata.mines.edu/nighttime_light/monthly/v10/). Considering the temporal overlap of NPP-VIIRS data and DMSP-OLS data, the annual composite product was synthesized using the median of monthly composite products of 2013, which reduces the impact of noise and improves data quality.

(4) **EVI data for calculating EANTLI**

The EANTLI proposed by Zhuo *et al*.^21^ can reduce the saturation effect in urban centers. It can be expressed mathematically in the equation:

1 $$EANTLI=\frac{1+(nNTL-EVI)}{1-(nNTL-EVI)}\times {\rm{NTL}}$$

where *EVI* is the annual maximum value of EVI (enhanced vegetation indices), *NTL* is the DMSP-OLS nighttime light intensity, while *nNTL* indicates the normalized *NTL*. EVI data is provided by the MODIS MOD13A2 V6 product (https://lpdaac.usgs.gov/products/mod13a2v006/), containing 16-day vegetation index maps at a 1 km spatial resolution. Considering the inconsistency in spatial resolution, we resampled the MODIS EVI product to 30 arcsec resolution using bilinear interpolation.

(5) **Global urban data and socio-economic data**

Since the source of stable light at night is mainly artificial light in urban areas, extracting city-wide is one of the most common applications of NTL data. To further validate the potential of the CCNL dataset for urban studies, the global urban boundaries dataset and global urban land dataset were used to evaluate the quality of CCNL. Li *et al*.^33^ developed an automatic delineation framework to generate a 30 m resolution global urban boundaries (GUB) dataset in seven representative years (i.e., 1990, 1995, 2000, 2005, 2010, 2015, and 2018) using 30 m global artificial impervious area (GAIA) data^34^. The GUB dataset can be freely downloaded from http://data.ess.tsinghua.edu.cn/gub.html. He *et al*.^35^ proposed a fully convolutional network (FCN) and employed it to extract the global urban land (UrbanLand^36^) in 1992, 1996, 2000, 2006, 2010, and 2016 using multi-source remotely sensed data, with an average overall accuracy (OA) of 90.9% and an average kappa value of 0.47. The UrbanLand dataset can be accessed from https://doi.pangaea.de/10.1594/PANGAEA.892684. The socio-economic data, including GDP, electricity, and population data for world countries from 1992 to 2013, were provided by World Bank (https://data.worldbank.org/indicator).

### Framework

**Fig. 2:**
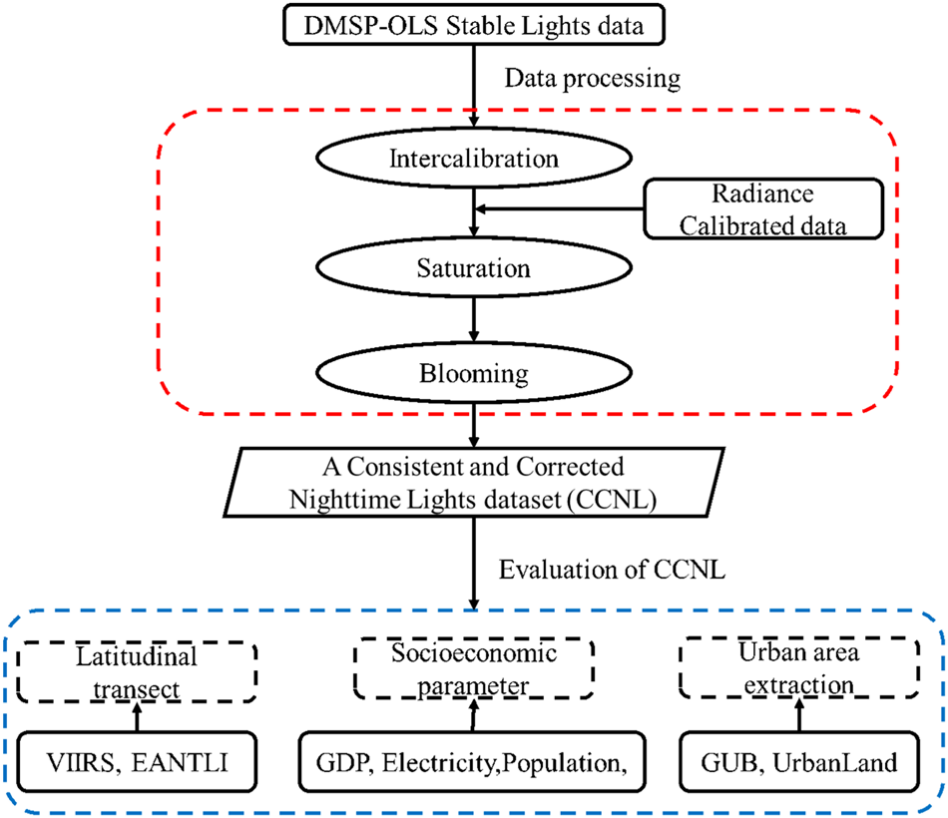
Flowchart of CCNL dataset production and evaluation.

The DMSP-OLS NTL product suffers from three main problems, i.e., interannual inconsistency, saturation, and blooming effect, which will affect the accuracy of urban extraction and the estimation of the social-economic indexes. The study first adopted three correction methods to rectify interannual inconsistency, saturation, and blooming effects, as illustrated in Fig. 2. We then used auxiliary datasets to evaluate the CCNL dataset in terms of the transect, socio-economic statistics, and urban extraction.

#### Interannual inconsistency correction

**Fig. 3:**
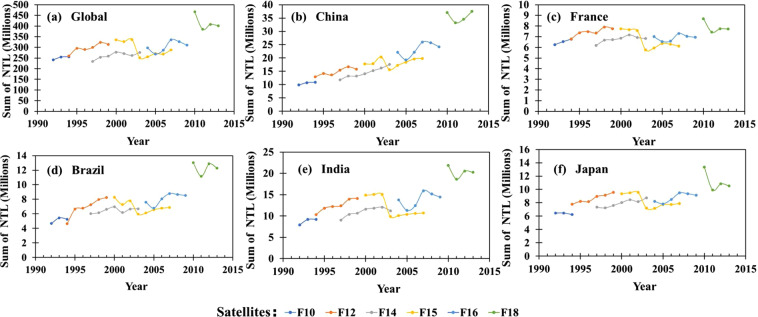
Total NTL DN value of DMSP-OLS stable light product worldwide and across countries.

As shown in Fig. 3, there is an apparent interannual inconsistency in the sum of NTL DN values at global and national scales. For example, we can observe a significant decrease in F15 satellite data from 2002 to 2003 in all regions, with this phenomenon present throughout the stable light products. Penny *et al*.^19^ proved that globally applicable NTL calibration minimizes interannual bias to a higher extent than regionally applicable NTL calibrations. Zhang *et al*.^37^ pointed out that Wu^30^’s and Zhang^31^’s respective methods have good performance in global-scale applications. Based on geographical location, uniform range distribution, and distance from the mainland, Wu *et al*.^30^ selected Mauritius, Puerto Rico, and Okinawa as PIF regions, with the radiance calibrated data in 2006 as the reference image and the power function model as the correct model. Because of the simplicity of Wu’s method, we selected it for interannual correction of the original images in this study. The regression model is as follows:

2 $$D{N}_{c}+1=a\times {\left(D{N}_{m}+1\right)}^{b}$$

where *DN*_*c*_ is the pixel value after correction, *DN*_*m*_ is the original DN value, a and b are the unknown coefficients in the model. Wu *et al*.^30^ provided correction factors for the years 1992 to 2010, and we calculated the correction factors for the remaining years according to their method. The model coefficients are shown in Table 2. When there are two interannual calibration results for the same year, for cases F142000 and F152000, we took the average of these two results as the final result.

**Table 2:** Coefficients of Interannual correction model.

Image	a	b	Image	a	b
F101992	0.8959	1.0310	F152001	0.8678	1.0646
F101993	0.6821	1.1181	F152002	0.7706	1.0920
F101994	0.9127	1.0640	F152003	0.9852	1.1141
F121994	0.4225	1.3025	F152004	0.8640	1.1671
F121995	0.3413	1.3604	F152005	0.5918	1.2894
F121996	0.9274	1.0576	F152006	0.9926	1.1226
F121997	0.3912	1.3182	F152007	1.1823	1.0850
F121998	0.9734	1.0312	F162004	0.7638	1.1507
F121999	0.9662	1.0265	F162005	0.6984	1.2292
F141997	1.2133	1.0189	F162006	0.9028	1.1306
F141998	0.9824	1.1070	F162007	0.8864	1.1112
F141999	1.0347	1.0904	F162008	0.9971	1.0977
F142000	0.9885	1.0702	F162009	1.4637	0.9858
F142001	0.9282	1.0928	F182010	0.8114	1.0849
F142002	0.9748	1.0857	F182011	0.9021	1.0678
F142003	0.9144	1.1062	F182012	1.0825	1.0066
F152000	0.8028	1.0855	F182013	0.9426	1.0672

#### Saturation correction

Recently, Hu *et al*.^23^ proposed a saturation correction method based on regression model and radiance-calibrated NTL data (SARMRC) by using discrete radiance calibrated data to correct the saturation effect in the annual stable light data. Compared to other saturation correction methods, SARMRC methods do not require different types of data and perform very well, so we used the SARMRC method for saturation correction on a global scale. We identified the region with a DN value of 63 as the saturation region. Sample pixels were selected based on the difference between the DMSP/OLS stable light data and the radiance calibrated data from neighboring years. Saturation zone DN values can be obtained from the corresponding area of the radiance calibrated data and the logarithmic model. The specific equation is as follows:

3 $${DN}_{LM}=a\times {\rm{\log }}\left({DN}_{R}\right)+b$$

where *DN*_*LM*_ is the DN value corrected by the logarithmic model, *DN*_*R*_ is the radiance calibrated data DN value of the saturated zone, *a* and *b* are the coefficients of the regression model. Some stable light data have radiance calibrated data from two adjacent years, for example, the stable light data in 2008 can be corrected by the data from 2006 or 2010, respectively. Therefore, for this type of stable light data, we used the weighted average of its two correction results as the final correction result, as follows:

4 $$D{N}_{LMDA}=\frac{{R}_{1}^{2}}{{R}_{1}^{2}+{R}_{2}^{2}}\times D{N}_{LM1}+\frac{{R}_{2}^{2}}{{R}_{1}^{2}+{R}_{2}^{2}}\times D{N}_{LM2}$$

where *DN*_*LMDA*_ is the double-year adjusted DN value, *DN*_*LM*1_ and *DN*_*LM*2_ are the correction results obtained from the radiation correction data of different years, ${R}_{1}^{2}$ and ${R}_{2}^{2}$ are the correlation coefficients between the stable light data and the radiation correction data, respectively.

#### Blooming effect correction

We chose the SEAM model proposed by Cao *et al*.^26^ which does not require auxiliary data and works well. SEAM model assumes that a pseudo light pixel (i.e., a bright pixel adjacent to the background) should have no light, and its value is contributed by the blooming effect of other bright pixels around it. The blooming effect can be quantitatively described by a spatial response function with pseudo light pixels as samples, and the specific equation is as follows:

5 $$R{\prime} =a\times {\sum }_{i=1}^{N}\frac{{R}_{i}}{{d}_{i}^{2}}+b$$

where *R*′ is the value of brightness change due to the blooming effect, *R*_*i*_ represents the pixel value of the neighboring pixels in the moving window, *N* is the number of neighboring pixels, *d*_*i*_ is the Euclidean distance from the pseudo-pixel, a and b are coefficients describing the blooming effect. Using the pseudo-pixel as a sample, the coefficients *a* and *b* were obtained by regression analysis. After obtaining the coefficients, we can estimate the luminance value due to the blooming effect for any bright pixel. The final result was obtained by subtracting the brightness value caused by the blooming impact from the original brightness value. To alleviate saturation’s influence on blooming effect correction, we performed the saturation effect correction before blooming effect correction on DMSP-OLS NTL images^27^.

#### Calculation platform

Google Earth Engine (GEE) is a cloud-based platform for geospatial analysis with many publicly available image datasets^38^. GEE has played an essential role in the fields of resource mapping, disaster monitoring, public health, and environmental protection^39–42^. We leveraged the superb computing power and the rich public datasets of the GEE platform to complete the entire data production process.

## Data Records

The consistent and corrected nighttime light dataset (CCNL^43^) from DMSP-OLS data (CCNL 1992–2013) in the WGS84 coordinate system with a spatial resolution of 30 arcsec (~1000 m) can be freely accessed at the Zenodo repository (10.5281/zenodo.6644980), which is stored as the GeoTIFF format (~300 MB) for each year. The current version of products spans the globe from 75 N latitude to 65 S.

## Technical Validation

The CCNL dataset was produced to apply to Spatio-temporal analysis at both global and local scales, overcome important problems in DMSP data and unlock the potential of NTL products. We evaluated the quality of CCNL in three aspects. Firstly, the spatial information of the CCNL dataset at the local scale was measured by cross-sectional analysis. Secondly, the effectiveness of city-scale extraction from the CCNL dataset was assessed at spatial and temporal scales. Finally, the performance of CCNL data in the temporal analysis was verified at a large scale by using socio-economic data such as GDP, population, etc.

(1) **Comparison of transects on NTL images**

**Fig. 4:**
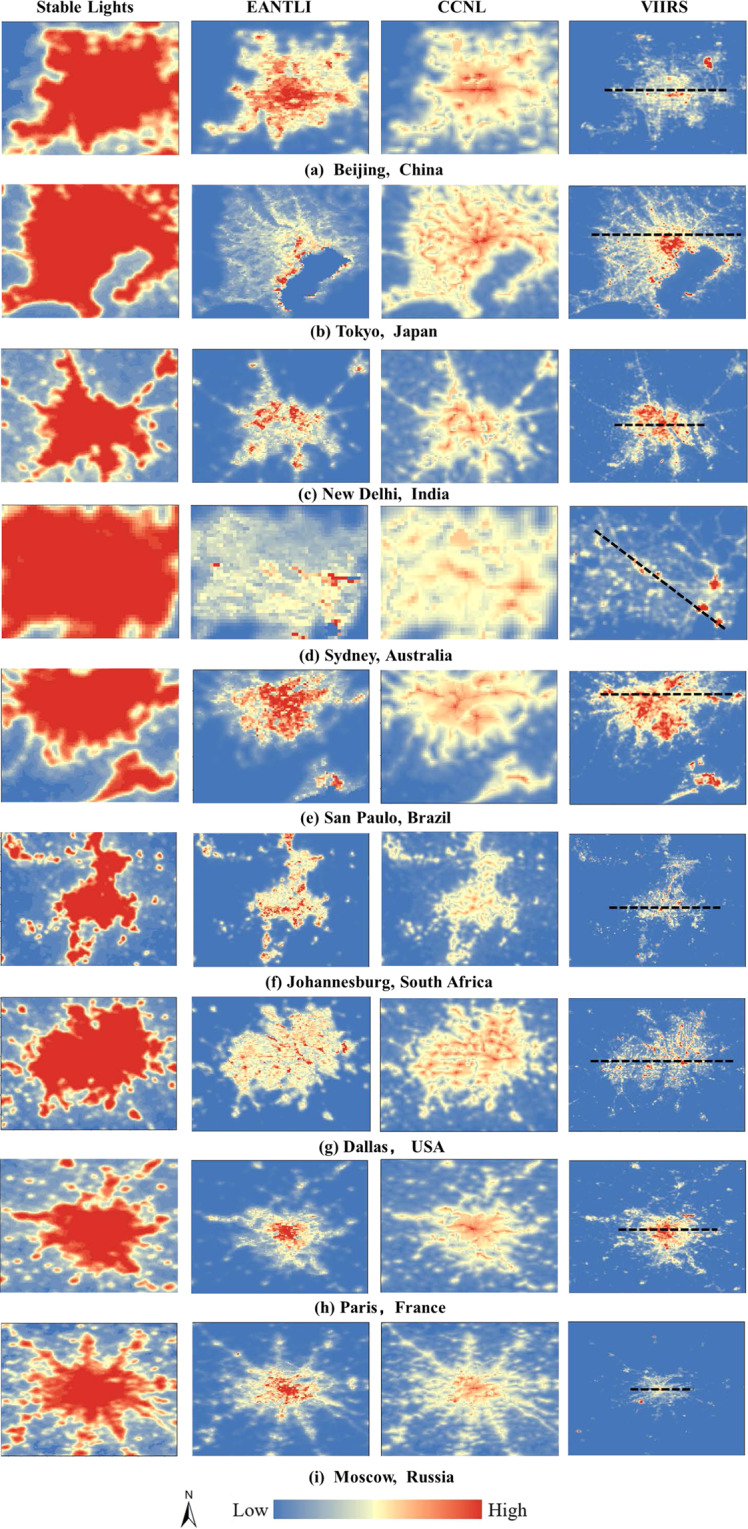
Comparison of nighttime light spatial distribution model between the DMSP-OLS stable light, EANTLI, CCNL, and NPP-VIIRS.

It is well known that the DMSP-OLS stable light data suffers from severe saturation and blooming effect problems, which seriously affect the application of the data. The method we proposed in this research can effectively address these challenges. We compared the stable light data, EANTLI, CCNL, and NPP-VIIRS data by visual and randomly selected data transects to evaluate the quality of the produced CCNL data. Nine cities around the world were selected to assess the quality of CCNL data, i.e., Beijing, Tokyo, New Delhi, Sydney, Sao Paulo, Johannesburg, Dallas, Paris, and Moscow (Fig. 4). These cities were selected because of their large urban extents, dramatic spatial variabilities of NTL, and the uniformity and representativeness of their global distribution.

Visually, NPP-VIIRS data can monitor the spatial pattern of nighttime lights in urban interiors thanks to its high spatial resolution and large dynamic range, which can be considered as ‘ground truth’. The EANITLI data mitigates blooming and saturation effects by combining nighttime light data and enhanced vegetation indices (EVI). The CCNL dataset produced in this research recovers the light intensity values in saturated regions by taking advantage of the radiance calibrated data. As shown in Fig. 4, we can observe roads and landmarks in CCNL and EANTLI. For example, one can see Beijing’s famous Chang’an Street and Tokyo’s complex road network.

**Fig. 5:**
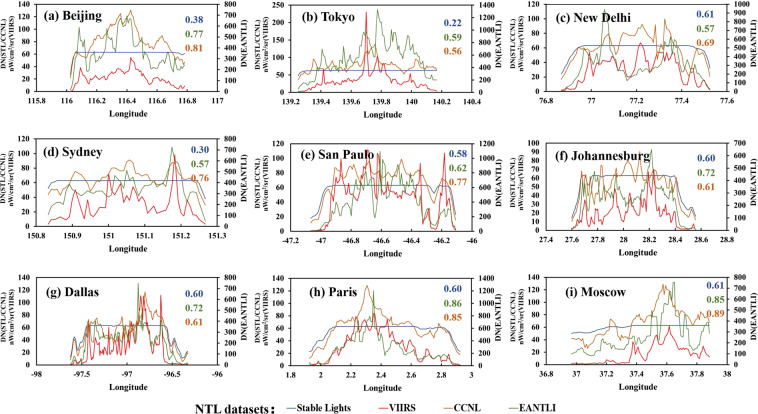
The DN curves of the transects for the nine selected cities. The dash lines on NPP-VIIRS images (Fig. 4) show the locations of the transects. The figures (colours corresponding to the legend) at the top right are the correlation coefficients (R) between the NTL datasets and the NPP-VIIRS data.

We selected a random latitude in each city’s center and extracted the latitude transects for each dataset. The NPP-VIIRS data were taken as the reference data, and the correlation coefficients R with other data were calculated separately. In the unsaturated region, the data show a similar pattern of variation. While in the saturated area, the DMSP-OLS stable light data reaches a maximum value of 63 and remains constant due to the saturation effect, which makes the data unable to provide spatial differences in lighting within the city. Both EANTLI and CCNL reduce the saturation effect with different magnitudes. The stable light dataset had the worst correlation, with a mean value of the correlation coefficient R of 0.49 (Fig. 5), while the mean values of R for the CCNL and EANTLI were 0.74 and 0.70, respectively. Paris has a spatial correlation coefficient R of up to 0.89 for CCNL. However, the R-values for CCNL are not always higher than EANTLI. In five of the nine cities, the R-values for CCNL are higher than EANTLI. CCNL has the lowest R-value of 0.56, while ENTLI is 0.57. The R-values of CCNL data are smaller than EANTLI data in some cities, but the R-values of the two datasets are very close.

(2) **Evaluation of urban extent extraction**

Urban area extraction is the most common application field for NTL data, as most of the stable lights at night come from artificial lights in urban areas. We extracted urban areas using a fixed threshold method and evaluated the results’ overall accuracy (OA). The GUB data^33^ was used as reference data for the qualitative analysis, and UrbanLand^35^ was used as reference data for quantitative evaluation.

**Fig. 6:**
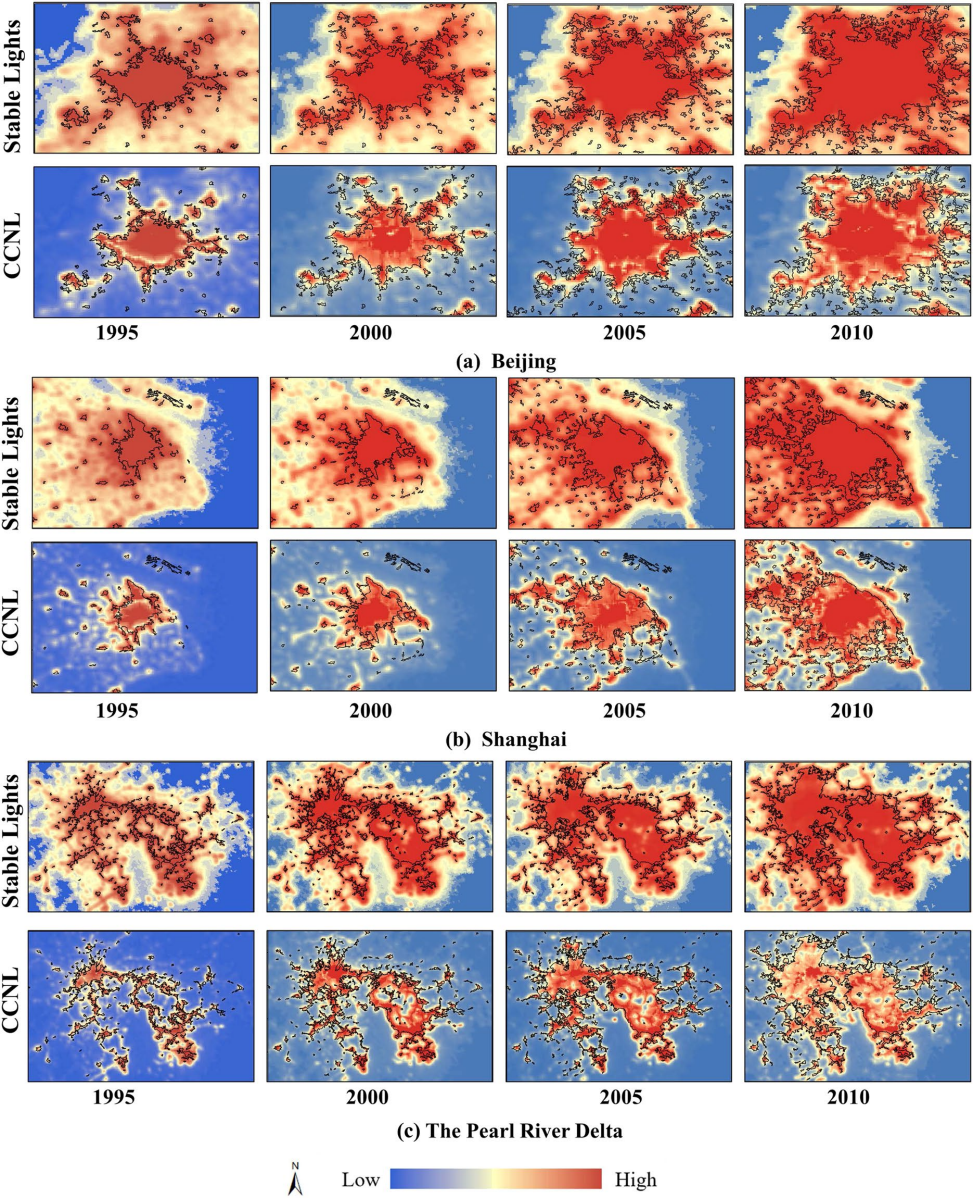
The spatial similarity of stable light data and CCNL with GUB data (black contours) in Beijing (**a**), Shanghai (**b**), and the Pearl River Delta(**c**).

The GUB dataset has a high resolution of 30 m, which can be used to verify the effectiveness of the CCNL dataset in urban extent extraction. We choose Beijing, Shanghai, and the Pearl River Delta region as visual test areas, as shown in Fig. 6, which have al experienced significant urban expansion over the past few decades. From the visual point of view, the light intensity values in the stable light data near the city boundary of the GUB data do not change significantly, and the blooming effect can be observed in different areas. Due to the severe spillover effect, the stable light data is not a good indicator of the true extent of the city. Compared with the stable light data, the spatial pattern of CCNL is more similar to GUB data. The values of CCNL vary with dramatic changes near city boundaries, which can be easily visualized. Even some small cities and towns can be observed, while only some tiny towns are not extracted, suggesting that the CCNL dataset is effective in eliminating the blooming effects.

We used the UrbanLand dataset as reference data to quantitatively characterize the effect of CCNL’s urban extraction. This study extracted city contours using a simple fixed-threshold method with thresholds derived from visual interpretation. The UrbanLand dataset extracts city limits with greater precision in six discrete years, i.e., 1992, 1996, 2000, 2006, and 2010. Table 3 shows the fixed thresholds for extracting city limits for different years in three cities), i.e., Paris, Tokyo, and Chicago, and the OA value for each dataset and each year, using the UrbanLand dataset as the reference dataset and fixed threshold method. Results show that the overall accuracy of the CCNL dataset for extracting city ranges reaches over 93% in Paris and Chicago and 88% in Seoul. In Paris, Seoul, and Chicago, CCNL’s OA increased by 1.94%, 1.43%, and 1.20%t, respectively, compared to the stable light data.

**Table 3:** Overall accuracy of city extent extracted by a fixed threshold method.

Year	Paris		Seoul		Chicago	
	Stable	CCNL	Stable	CCNL	Stable	CCNL
1992	89.22%	92.87%	90.56%	90.45%	93.75%	93.89%
1996	91.71%	93.75%	90.17%	89.20%	92.99%	94.15%
2000	92.19%	94.31%	88.56%	90.82%	92.92%	94.53%
2006	93.07%	94.60%	83.93%	87.53%	92.65%	93.30%
2010	92.90%	93.27%	80.75%	83.14%	89.76%	92.19%
Average	91.82%	93.76%	86.79%	88.23%	92.41%	93.61%

(3) **Characteristics of urbanization**

**Fig. 7:**
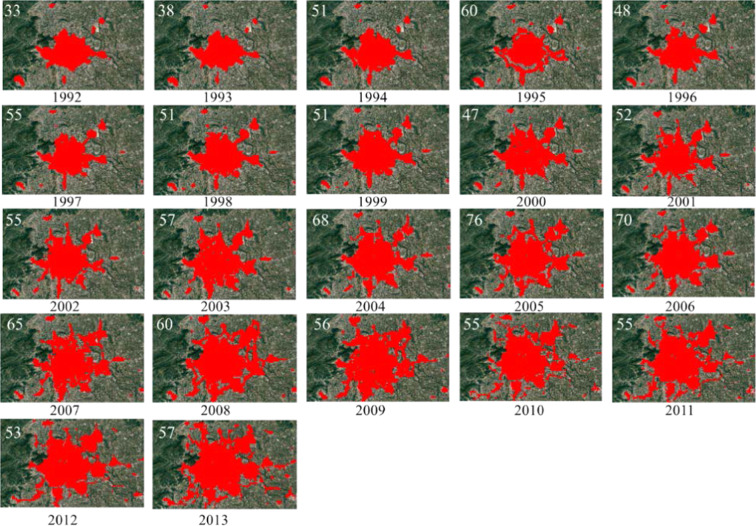
Results of Beijing city extent extracted (red) using fixed threshold (white number) method. The background images are from Google Earth.

CCNL provides 22 years of NTL sequence data from 1992 to 2013. Cities with significant expansion were selected as research subjects to analyze the role of the CCNL dataset in the urbanization process. The screening results showed that most eligible cities were concentrated in developing countries. For example, China’s Shenyang, Beijing, Zhengzhou, Xi’an, Shanghai, Changsha, Chengdu, and Guangzhou have significant urban expansion between 1992 and 2013. In Southeast Asia, many cities have similar expansion characteristics, such as Hanoi and Ho Chi Minh City in Vietnam and Kuala Lumpur in Malaysia. Beijing was chosen to verify the potential of CCNL in temporal analysis. The urban areas extracted using the fixed threshold method are shown in Fig. 7. In Fig. 7, we can observe Beijing’s urbanization, the city’s continuous expansion, and the suburbs’ development. The UrbanLand dataset was used as reference data to calculate the OA of the extracted results (Table 4). The OA is greater than 90%, with an average value of 94.2%.

**Table 4:** Overall accuracy of Beijing extent extracted in different years.

Year	Threshold	OA
1992	33	97.59%
1996	48	95.69%
2000	47	94.63%
2006	70	93.36%
2010	50	90.19%

(4) **Performance of CCNL time series**

The density of the use of regional lighting facilities can, to a certain extent, reflect the economic situation, energy consumption, and population of the region, and a large number of statistical studies have proved that there is a high correlation between the intensity of nighttime light and this socio-economic data^6–8^. CCNL provides the time series of NTL from 1992 to 2013 and reduces the temporal inconsistencies in the original time series through the relative correction methods. To demonstrate the effectiveness of the interannual correction method, we selected ten countries and performed a correlation analysis using economic, population, and energy consumption data from the World Bank (Table 5). These ten countries were selected from developed and developing countries on different continents.

**Table 5:** The correlation coefficients (R) between NTL intensity and GDP, electricity, urban population, and total population in the 10 selected countries.

Country	Data	GDP	Electric power consumption	Urban population	Total population
Japan	Stable Lights	0.57	0.21	0.31	0.24
	CCNL	0.20	0.88	0.54	0.80
UK	Stable Lights	−0.15	−0.37	0.11	0.12
	CCNL	0.38	0.51	0.17	0.18
Germany	Stable Lights	0.39	0.36	0.33	−0.22
	CCNL	0.53	0.59	0.72	0.12
France	Stable Lights	0.63	0.57	0.74	0.73
	CCNL	0.75	0.86	0.84	0.84
South Korea	Stable Lights	0.79	0.83	0.83	0.84
	CCNL	0.81	0.89	0.95	0.93
China	Stable Lights	0.96	0.97	0.94	0.88
	CCNL	0.93	0.98	0.99	0.96
India	Stable Lights	0.88	0.89	0.83	0.81
	CCNL	0.91	0.93	0.94	0.94
Thailand	Stable Lights	0.85	0.81	0.81	0.74
	CCNL	0.87	0.92	0.89	0.87
Australia	Stable Lights	0.82	0.45	0.79	0.78
	CCNL	0.78	0.82	0.89	0.90
Brazil	Stable Lights	0.93	0.91	0.81	0.82
	CCNL	0.88	0.98	0.96	0.96

Regarding power consumption, the correlation coefficient (R) of CCNL is higher than that of stable light, and the average R-value is 0.84 and 0.56, respectively, which are greatly improved. The correlation coefficient between urban population and NTL intensity is higher than that of the total population because artificial lights at night are mainly concentrated in urban areas and less distributed in the suburbs. In terms of the total population and urban population, the average R-value of CCNL increased by 0.17 and 0.14, respectively. While in terms of GDP, CCNL performed even worse than the stable light data in some countries, possibly because the change in GDP data does not fully reflect the change in light intensity, especially in some developed countries. Another reason is that a global model for interannual correction may result in overcorrection in some regions.

## Usage Notes

Similar to DMSP-OLS stable light data, the pixel value of CCNL data is the digital number, not a physical quantity. The auxiliary data used to eliminate the saturation effect is the DMSP-OLS radiance calibrated dataset (1996–2010). For years beyond the period (1992–1995 and 2011–2013), the correction of a saturated region relies on the same data, and the processed data have the same spatial structure, which may not reflect the spatial change of the region. When using CCNL dataset for temporal change analysis, it is recommended to analyze the changes of sum of NTL values at regional or national scale (window size equivalent or over 200 pixels), while pixel scale and small statistical regions may have large fluctuations. This dataset can be used for monitoring human activities at local and global scales and for historical time series analysis.

## Data Availability

The source code for processing the DMSP-OLS Stable Light dataset to produce the CCNL dataset is available at 10.5281/zenodo.6100284^44^.
